# Transformative Leadership: Emergency Physicians Lead AOA and AMA

**DOI:** 10.5811/westjem.2015.10.28816

**Published:** 2015-11-16

**Authors:** Chadd K. Kraus

**Affiliations:** University of Missouri-Columbia, Department of Emergency Medicine, Columbia, Missouri

This was a historic summer in Chicago for emergency medicine. On July 18, 2015, John W. Becher, DO, became the 119^th^ president of the American Osteopathic Association (AOA), while a month earlier, on June 9, 2015, Steven J. Stack, MD, became the 170^th^ president of the American Medical Association (AMA). This is the first time that emergency physicians have led the two largest professional physician organizations in the United States, and the first time that an emergency physician has led the AMA. Together, these two groups represent more than 330,000 medical students, residents, and practicing physicians, with the AMA representing nearly 232,000 members^1^ and the AOA representing almost 110,000 osteopathic physicians and physicians-in-training.^2^

The election of emergency physicians to guide the AMA and AOA for the coming year is historic in the context of our specialty’s evolution in organized medicine and, more broadly, as a leader in population health. The biographies of Dr. Becher and Dr. Stack reflect this evolution. For several decades, Dr. Becher has been the chair of emergency medicine at the Philadelphia College of Osteopathic Medicine (PCOM), one of the oldest academic departments of emergency medicine. While he has been involved in organized medicine and emergency medicine leadership positions throughout his career, Dr. Stack is the youngest AMA president in 160 years.^3^ In the early days of our specialty, when a fledgling department of emergency medicine was being established at PCOM and a few other medical schools around the country and both MD and DO emergency physicians were working to establish specialty recognition and board certification, it would have been almost unimaginable that in a few short decades an emergency physician would become one of the youngest presidents in the history of the AMA. It is a testament to the growth and strength of our specialty and to the accomplishments of emergency physician leaders in the house of medicine.

The impact of emergency medicine on the leadership development of both Drs. Becher and Stack was evident in their inauguration speeches.^4^,^5^ Drs. Becher and Stack gave credit to the central role of their training and experiences as emergency physicians in preparing them to lead their respective organizations. They cited the ability to adapt quickly under unexpected circumstances, being prepared for the unexpected, the unique place of emergency medicine in caring for patients and communities in their most vulnerable times, and the team-centered nature of clinical care in the emergency department as ways that emergency medicine equipped them to be leaders in organized medicine. Finally, Drs. Becher and Stack stressed their roles as servant leaders working within larger teams, both in their clinical work and as presidents of the AOA and AMA. Dr. Stack put this leadership lesson succinctly: “The lesson from the pages of history could not be more clear: an empire built by one man will not stand. An empire built by many endures.”^5^

From a population health perspective, having emergency physicians at the helm of the AOA and AMA is a unique opportunity to impact U.S. healthcare for years to come, from how physicians are trained to how care is delivered to the presence of physicians in public policy discussions. Drs. Becher and Stack have discussed common themes during their tenure on the AOA and AMA boards of trustees and in their respective inauguration speeches: innovating medical education; meeting patient’s primary healthcare needs; and, advocating in the public policy and legislative arena.

The education and training of an adequate physician workforce is a priority for the AOA and the AMA.^6^–^8^ Specifically, both organizations have been part of larger stakeholder discussions of how to accelerate innovations and reforms in medical education that will prepare the physician of the future to engage in the challenges of a dynamic healthcare environment, and to encourage the longevity of those physicians once in practice. These transformations will occur in both undergraduate medical education and graduate medical education (GME), with a renewed focus on competency-based outcomes, a unified GME accreditation system on the horizon, and continuing threats to GME funding.

Supporting strategies that meet the primary healthcare needs of patients was also a theme of both inauguration speeches. Specifically, Dr. Stack pointed to the need to improve outcomes for the millions of Americans with pre-diabetes and hypertension.^5^ Dr. Becher highlighted the special role of the osteopathic profession in training primary care physicians.^4^ And both leaders, as emergency physicians, stressed the integrated teams and interdisciplinary focus necessary to provide optimal outcomes across the care continuum.

Lastly, they discussed the need for engagement in advocacy for public policies that allow physicians to provide care in a system that meets the “Triple Aim” of improving the patient experience, improving population health, and reducing costs.^9^ Among the issues raised by Dr. Becher and Dr. Stack include the transition to value-based reimbursement, meaningful use implementation, and the role of non-physician clinical providers.^7^,^10^,^11^

This is a historic year for emergency medicine as two of our specialty’s leaders guide America’s physicians during an era of healthcare transformation. The examples of Drs. Becher and Stack prove the unique preparation and skills emergency physicians bring to the challenges facing 21^st^ century healthcare.
